# Characterization of atherosclerotic plaques in blood vessels with low oxygenated blood and blood pressure (Pulmonary trunk): role of growth differentiation factor-15 (GDF-15)

**DOI:** 10.1186/s12872-021-02420-9

**Published:** 2021-12-17

**Authors:** G. A. Bonaterra, N. Struck, S. Zuegel, A. Schwarz, L. Mey, H. Schwarzbach, J. Strelau, R. Kinscherf

**Affiliations:** 1 Institute for Anatomy and Cell Biology, Department of Medical Cell Biology, University of Marburg, 35032 Marburg, Germany; 2 Department of Functional Neuroanatomy, University of Heidelberg, 69120 Heidelberg, Germany

**Keywords:** ApoE, Apoptosis, Atherosclerosis, GDF-15, Hypercholesterolemic mice, Macrophage, Pulmonary trunk

## Abstract

**Background:**

Growth differentiation factor (GDF)-15 is linked to inflammation, cancer, and atherosclerosis. GDF-15 is expressed in most tissues but is extremely induced under pathological conditions. Elevated serum levels are suggested as a risk factor and a marker for cardiovascular diseases. However, the cellular sources and the effects of GDF-15 on the cardiovascular system have not been completely elucidated including progression, and morphology of atherosclerotic plaques. Thus, this work aimed to characterize the influence of GDF-15 deficiency on the morphology of atherosclerotic plaques in blood vessels with low-oxygen blood and low blood pressure as the pulmonary trunk (PT), in hypercholesterolemic ApoE^−/−^ mice.

**Methods:**

GDF-15^−/−^ ApoE^−/−^ mice were generated by crossbreeding of ApoE^−/−^- and GDF-15^−/−^ mice. After feeding a cholesterol-enriched diet (CED) for 20 weeks, samples of the brachiocephalic trunk (BT) and PT were dissected and lumen stenosis (LS) was measured. Furthermore, changes in the cellularity of the PT, amounts of apoptosis-, autophagy-, inflammation- and proliferation-relevant proteins were immunohisto-morphometrically analyzed. Additionally, we examined an atherosclerotic plaque in a human post mortem sample of the pulmonary artery.

**Results:**

After CED the body weight of GDF-15^−/−^ApoE^−/−^ was 22.9% higher than ApoE^−/−^. Double knockout mice showed also an 35.3% increase of plasma triglyceride levels, whereas plasma cholesterol was similar in both genotypes. LS in the BT and PT of GDF-15^−/−^ApoE^−/−^ mice was significantly reduced by 19.0% and by 6.7% compared to ApoE^−/−^. Comparing LS in PT and BT of the same genotype revealed a significant 38.8% (ApoE^−/−^) or 26.4% (GDF-15^−/−^ApoE^−/−^) lower LS in the PT. Immunohistomorphometry of atherosclerotic lesions in PT of GDF-15^−/−^ApoE^−/−^ revealed significantly increased levels (39.8% and 7.3%) of CD68^ +^ macrophages (MΦ) and α-actin^ +^ smooth muscle cells than in ApoE^−/−^. The density of TUNEL^ +^ , apoptotic cells was significantly (32.9%) higher in plaques of PT of GDF-15^−/−^ApoE^−/−^ than in ApoE^−/−^. Analysis of atherosclerotic lesion of a human pulmonary artery showed sm-α-actin, CD68^+^, TUNEL^+^, Ki67^+^, and APG5L/ATG^+^ cells as observed in PT. COX-2^+^ and IL-6^+^ immunoreactivities were predominantly located in endothelial cells and subendothelial space. In BT and PT of GDF15^−/−^ApoE^−/−^ mice the necrotic area was 10% and 6.5% lower than in ApoE^−/−^. In BT and PT of GDF15^−/−^ApoE^−/−^ we found 40% and 57% less unstable plaques than ApoE^−/−^ mice.

**Conclusions:**

Atherosclerotic lesions occur in both, BT and PT, however, the size is smaller in PT, possibly due to the effect of the low-oxygen blood and/or lower blood pressure. GDF-15 is involved in atherosclerotic processes in BT and PT, although different mechanisms (e.g. apoptosis) in these two vessels seem to exist.

**Supplementary Information:**

The online version contains supplementary material available at 10.1186/s12872-021-02420-9.

## Background

The heart as the major component of the cardiovascular system (CVS), pumps blood into the pulmonary and systemic circulation. The right part of the heart receives deoxygenated blood from the peripheral tissues through the cava veins and pumps it into the pulmonary trunk (PT) by low-pressure pulmonary circulation. It is well known that the lumen of, *e.g.* carotid and coronary arteries may be stenosed due to atherosclerosis and their normal bloodstream is affected. However, the mechanisms of development of atherosclerotic lesions in the system of pulmonary arteries, i.e. low blood pressure vessels and low partial pressure of oxygen are still unclear. In general, atherosclerosis is an inflammatory disease, characterized by the chronic accumulation of inflammatory cells, storage of lipids and fibrous component in the innermost layer of the arterial wall [1]. Increased serum low-density lipoprotein (LDL) cholesterol in the form of modified/oxidized LDL (oxLDL) is believed to play a key role during all stages of the disease by regulating the expression of chemokines and proinflammatory cytokines in the arterial wall [1]. Pulmonary artery atherosclerosis is a frequent autopsy finding, associated with different clinical conditions, and an important predictor of aortic atherosclerosis, ventricular hypertrophy and pulmonary embolization [2, 3]. In this context, pulmonary embolism (PE) represents the occlusion of the pulmonary artery branches by a thrombus (blood clot) that has traveled from elsewhere through the bloodstream. PE is a common, lethal disease with considerably high morbidity and mortality [4]. Atherosclerotic lesions in the arterial tree often occur especially at bifurcations, i.e. at sites exposed to turbulent blood flow leading to mechanical shear stress on the vessel wall that affects the endothelial cell homeostasis by e.g. pro-inflammatory activation [3]. Interestingly, lesions do not develop in veins under normal environment of low pressure and high flow, but when veins are used as arterial bypass, e.g. aortocoronary venous bypass, they may develop atherosclerotic lesions, probably because they are subjected to high pressure [5, 6]. The precise mechanisms leading to the development and progression of atherosclerotic lesions still remain largely unknown, but the regulation by growth factors may have an impact in lesion development. This is further supported by the observation that growth factor concentrations are frequently increased under pathological conditions and therefore giving information on the severity of the disease [7]. However, there are limited data on the use of biomarkers in predicting morbidity in populations of patients e.g. with atherosclerosis or acute PE. In this context, growth differentiation factor-15 (GDF-15) is linked to e.g. cardiovascular diseases and is related to mortality in older adults [8, 9]. Recent data indicate that in patients with acute PE, elevated concentrations of GDF-15 amongst others, are helpful to identify patients at risk of death during the acute phase of PE [10]. Under normal conditions, GDF-15 is only weakly expressed in most tissues [11], which are protected from inflammation and lesion development [12]. GDF-15 is a distant and divergent member of the transforming growth factor (TGF)-β superfamily [11, 13–15] with characteristics that most recently suggested GDF-15 as a potent biomarker for cardiac events and/or atherosclerotic diseases [16, 17]. Previously, we have shown that GDF-15 is a factor implicated in several pathophysiological processes including autophagy, inflammation, chronic vascular diseases, cancer, ischemia, and atherosclerosis [18–21]. However, GDF-15 is strongly upregulated under conditions associated with cellular stress such as tissue hypoxia/hyperoxia, inflammation, and oxidative stress [22–25]. Moreover, GDF-15 significantly contributes to subclinical coronary heart disease (CHD) independently of established cardiovascular disease (CVD) risks [26] and has been frequently associated with CVD [27]. Interestingly, hypoxia contributes to the formation of many pathologies of the blood vessel wall, like atherosclerosis, aortic aneurysms, pulmonary artery stenosis, and chronic venous disease [25, 28, 29]. In this context, elevated GDF-15 levels have been recently found during early chronic obstructive pulmonary disease (COPD) [29–32]. Moreover, GDF-15 has a potentially protective role for endothelial cells by promoting the activation of HIF-1 (hypoxia-inducible factor-1), which has been found in human pulmonary microvascular endothelial cells and umbilical vein endothelial cells (HUVEC) under hypoxia [33, 34]. Hypoxia of the vascular wall may be caused by inadequate oxygenation or increased cellular oxygen demand triggered by shear stress and increased hydrostatic pressure [35]. Thus, induction of GDF-15 expression by hypoxia and shear stress in combination with its effects on cell proliferation and apoptosis suggests a functional role in pulmonary endothelial cells and thereby in the pathobiology of complex vascular lesions in pulmonary arterial hypertension (PAH) [33, 36]. However, the cellular tissue sources, as well as detailed functional effects of GDF-15 in the CVS, have not been completely elucidated. Several human studies associate GDF-15 levels with CVD as, acute myocardial infarction [37], hypertensive patients [38] and hypertensive left ventricular hypertrophy [39]. However, after prolonged hyperoxia and consequent lung injury, GDF-15 mRNA expression was also markedly induced and found up-regulated in the lungs of patients with PAH [22, 33]. Moreover, plasma GDF-15 levels in hypertensive patients with left ventricular hypertrophy (LVH) were higher than those of hypertensive patients without LVH [40]. Additionally, in hypertensive patients a positive correlation between plasma GDF-15 levels and LVH was found, suggesting that GDF-15 may be involved in the development of LVH hypertension [40]. A recent study has also demonstrated elevated serum levels of GDF-15 in patients with idiopathic pulmonary arterial hypertension (IPAH) [41].

In summary, GDF-15 seems to be involved in orchestrating atherosclerotic lesion progression in arteries [20, 42]. Thus, this work aimed to characterize the influence of GDF-15 deficiency on the development, progression, and morphology of atherosclerotic plaques in blood vessels with low-oxygen blood and low blood pressure as the PT, in hypercholesterolemic ApoE-knockout mice. Additionally, it was examined whether atherosclerotic changes exist in a human post mortem sample of the pulmonary artery.

## Methods

### Animals

GDF-15 knockout/lacZ knock in (GDF-15^−/−^) mice [43] were crossbred with ApoE knockout (ApoE^−/−^) mice (Charles River, Sulzfeld, Germany), were housed 4 to 5 in each cage under the same conditions, with dark–light cycles of 12 h and constant temperature of 24 ± 2 °C with ad libitum access to food and water in cages with a minimum area of 100 cm^2^ per animal according the GV-SOLAS (Committee for Animal Welfare Laboratory animal husbandry, August 2014. Link: http://www.gv-solas.de/fileadmin/user_upload/pdf_publikation/Tierhaltung/hal_201408Tiergerechte-Haltung-Maus.pdf) with appropriate environmental enrichment. An approval or permission from the farm owner to use the animals was not necessary.

Thus, male GDF-15^−/−^/ApoE^−/−^- and ApoE^−/−^-mice, strain C57BL/6 were used for all investigations. All animal experiments were approved by the Regierungspräsidium Karlsruhe (35–9185.81/G-99/06) and the local authorities at the University of Heidelberg and were done in compliance with the regulations for animal studies at the University of Heidelberg. This investigation conforms to the Guide for the Care and Use of Laboratory Animals (8th edition), 2011 [44]. All animal studies were performed in compliance with the German laws relating to the conduct of animal experimentation. Our manuscript adheres to the ARRIVE guidelines (http://www.nc3rs.org. uk/page.asp?id = 1357) for the reporting of animal experiments.

### Animal study and blood samples

After 9 weeks, the male mice were fed for 20 weeks with or without cholesterol-enriched diet (CED, TD.88137; Harlan Teklad, Madison, WI), 15.2% kcal protein, 42.7% kcal carbohydrates and 42.0% kcal fat (0.2% cholesterol). The groups of 5 mice were randomly assigned. Body weight and blood samples were taken before and after CED. Animals were deeply narcotized by inhalation with diethyl ether saturated air in a narcosis chamber until plane III or IV of deep tolerance stage III of Guedel was reached, according to GV-SOLAS recommendation (https://www.uwindsor.ca/animal-care-committee/sites/uwindsor.ca.animal-care-committee/files/module-10.pdf). A 15 ml falcon tube with a swab, soaked with diethyl ether, was used as a head mask to guarantee a deep narcosis stage during the perfusion procedure. The abdomen, thorax and the right cardiac auricle were opened quickly (within 30 s). The outflowing blood was collected from the thoracic cavity, at this time point, death occurred. The vascular system was perfused with a solution consisting of 38°-39 °C warmed phosphate PBS with 5 Ul/ml heparin (Liquemin® 25,000 Ul/5 ml, Roche, Grenzach, Germany) afterward the brachiocephalic trunk (BT) and pulmonary trunk (PT) were removed.

The blood samples were heparinized (0.25 I.U./ml, Roche) and plasma was separated after 10 min centrifugation (650 × g). Plasma lipids (total cholesterol and triglycerides) were analysed using via enzymatic endpoint method (Randox Lab., Crumlin, UK) and GPO-PAP method (glycerol- 3-phosphate oxidase–peroxidase; Randox Lab.), respectively, according to the manufacturer’s instructions.

### Genotyping

Genomic DNA was isolated, according to the manufacturer’s instructions (DNA Extraction Solution, Epicentre Biotechnologies, Madison, USA). Transgenic positive animals were identified by polymerase chain reaction (PCR) of genomic tail DNA, using intron spanning oligonucleotides [20].

### Morphometry and immunohistology

For morphometric and immunohistological investigations, the BT and PT from mouse genotypes were fixed in 4% (v/v) paraformaldehyde (PFA) / Phosphate Buffered Saline (PBS), as well all post mortem sample of the left pulmonary artery (Additional file 1: Fig. S1) extracted from donated bodies of the Institute for Anatomy, fixed in 4% paraformaldehyde / 96% ethanol. The use and examination of the pulmonary arteries were approved by the local Ethics committee of the Medical Faculty of the Philipps University of Marburg (AZ: Study 80/21).

After fixation, the vessels were washed with several changes of PBS and dehydrated by an ascending series of alcohols (50%, 70%, 90%, 100%, and isopropanol). The alcohol was then removed from the tissue by storing it in cedar oil for several days. The material was then left in hot paraffin (60° C) for 5–6 h and after hardening subsequently cut using a microtome. The extent of atherosclerotic plaques was morphometrically measured by computer-assisted morphometry. Immunohistochemistry was routinely performed according to methods described earlier [20, 45].

Sodium citrate retrieval was performed using a microwave at 600 W (2 min) and 10 min by 360 W (CD68; Ki67; APG5L/ATG, COX-2 and IL6), or using proteolytic digestion Pepsin/0.01 M HCL (0.4%) by RT 20 min (α-Actin). Immunoreactions were achieved with the following antibodies: Polyclonal (pc) rabbit anti-mouse smooth muscle (sm-) α-actin, pc rabbit anti-mouse APG5L/ATG, pc rabbit anti-mouse Ki67, COX-2 and IL-6 (Abcam, Cambridge, UK); monoclonal (mc) mouse anti-human CD68 (Dako, Hamburg, Germany), as well as mc rat anti-mouse CD68 (AbD Serotec, Düsseldorf, Germany).

Single staining was performed after incubation of the sections with the primary antibody and thereafter with biotinylated rabbit anti-Maus (Dianova GmbH, Hamburg, Germany), goat anti-rabbit horseradish peroxidase (HRP)-conjugated (Linaris GmbH, Mannheim, Germany); sheep anti-Digoxigenin-HRP conjugated. Vectastain ABC-Kit (Vector Laboratories Inc., Burlingame, USA) and 3,3′-diaminobenzidine (DAB, Merck/Sigma-Aldrich Chemie GmbH, Munich, Germany) were used as detection system.

Necrotic cores (Nc) were defined as the lesion area absent of nuclei [47, 48] with modifications. To classify lesions as stable and unstable some characteristic plaque features were evaluated: Quantification of Nc (unstable lesion with large Nc was defined as occupying > 10% and stable < 10% of the total surface plaque, because necrotic areas of the lesions of the PT reached a minimum area of 8.0% and a maximum area of 14.5%, the mean between both extremes was taken as a limit, i.e. 10% (exactly 11.2% ± 1.2). Consequently, using the 10% Nc and disruption/rupture of the internal elastic lamina (media degeneration), the number of stable and unstable plaques was quantified in BT and PT to get accordingly informations concerning comparison of BT and PT.

### Detection of DNA fragmentation (TUNEL)

DNA fragmentation, a characteristic of apoptotic cells, was analysed on 4% PFA-fixed cryo cross sections by the TUNEL (TdT-mediated dUTP nick end labeling) technique, using the ApopTag kit (Oncor, Heidelberg, Germany) as previously described [48].

### Statistical analyses

All statistical analyses were performed with SigmaPlot 12® (Systat Software Inc., San José, USA) by comparing ApoE^−/−^ and GDF15^−/−^ApoE^−/−^ mice or BT vs PT. Statistical significance was determined by the unpaired 2-tailed Student’s t-test. The Mann–Whitney U rank-sum W test was applied when data failed normality and/or equal variance test. Thus, Shapiro–Wilk normality test and Brown–Forsythe equal variance test were applied. When appropriate, statistical significance was determined by one-way analysis of variance (ANOVA). Results are presented as a means + standard error of the mean (SEM). P values of less than 0.05 (p < 0.05) were considered as statistically significant.

## Results

### Body weight and plasma lipid

**Fig. 1:**
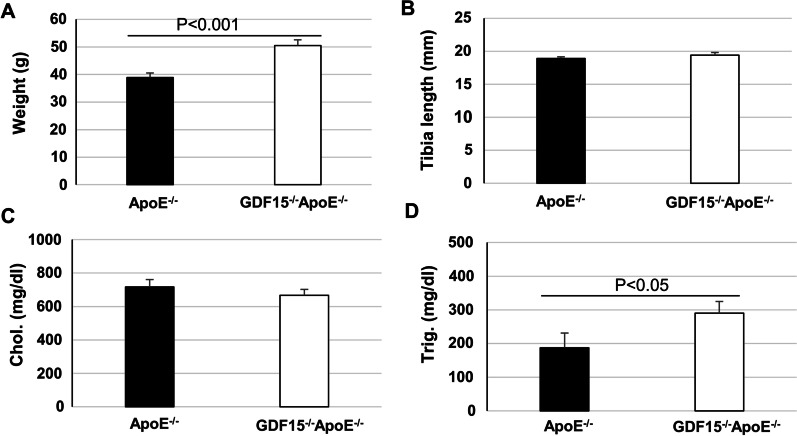
Effect of 20 weeks CED on body weight (**A**), tibia length (**B**), total (**C**) plasma cholesterol (Chol.) and (**D**) triglycerides (Trig.) of ApoE^−/−^ and GDF15^−/−^ApoE^−/−^ mice. Data are expressed as mean + SEM, n = 4–5

After 20 weeks CED the body weight of adult mice GDF-15^−/−^ApoE^−/−^ was significantly (p < 0.001) 22.9% higher than that of ApoE^−/−^ animals, whereas the tibia length was similar in both genotypes (Fig. 1A, B). To assess, whether the gain of weight in the absence of GDF-15 relates to lipid metabolism, total plasma cholesterol, and triglyceride levels were determined: After 20 weeks CED, plasma triglyceride significantly (p < 0.05) increased by 35.3% in GDF-15^−/−^ApoE^−/−^ mice compared with ApoE^−/−^ mice, whereas plasma cholesterol levels were similar in both genotypes (Fig. 1C and D).

### Cellular composition/morphology of atherosclerotic plaques

**Fig. 2:**
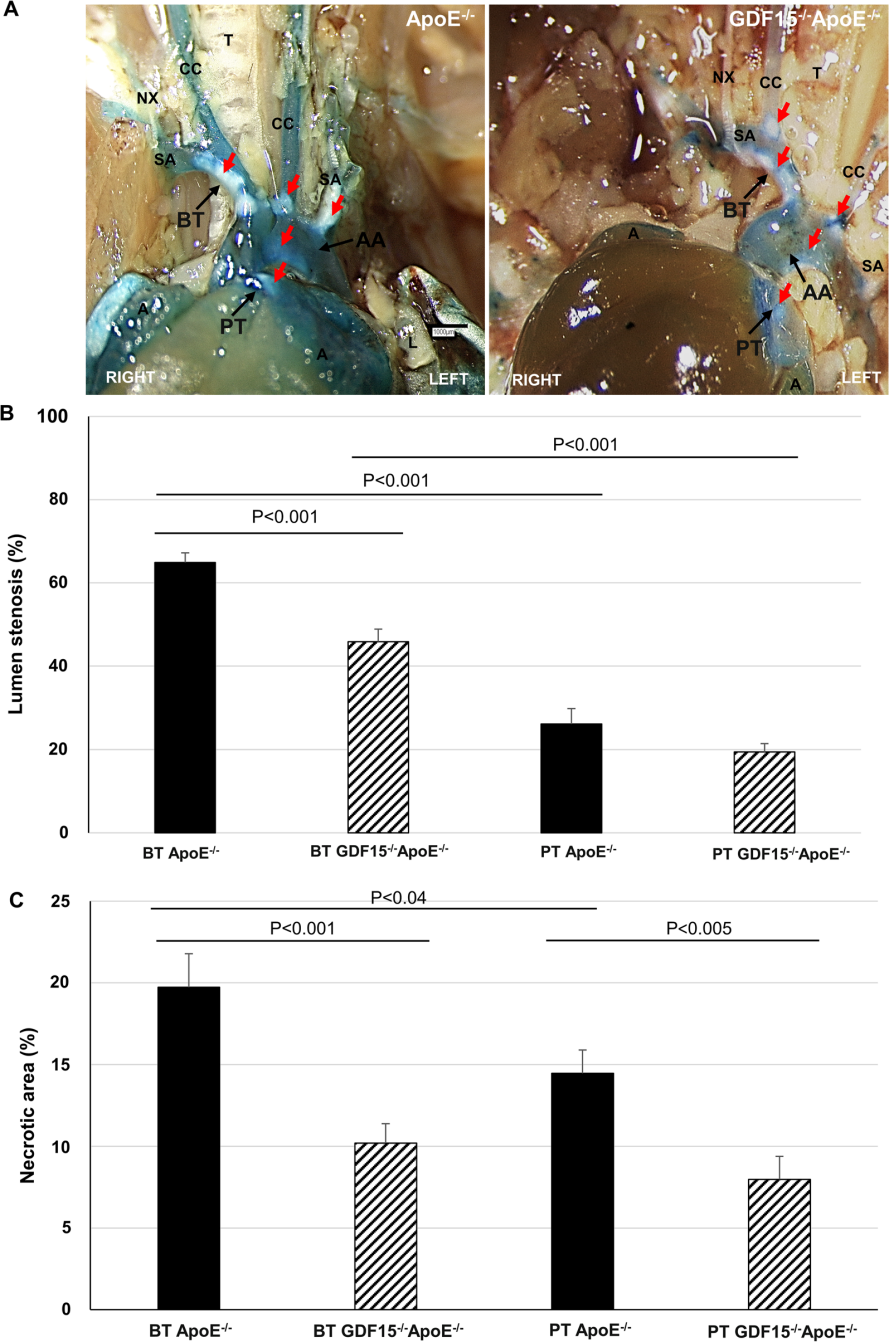
Effect of GDF-15 deficiency on lumen stenosis and necrotic core (Nc) areas in brachiocephalic trunk (BT) and pulmonary trunk (PT) of ApoE^−/−^- and GDF15^−/−^ApoE^−/−^ mice after 20 weeks CED. **A** Open chest photos: A, auricula; AA, aortic arch; CC, common carotid artery; NX, vagus nerve; SA, subclavian artery; T, trachea. Red arrows: atherosclerotic plaques. Scale bar: 1000 µm. Percentage of lumen stenosis (**B**) and Nc area (**C**). Data are expressed as mean + SEM, n = 4–10

Stenosis of blood vessels due to development of atherosclerotic plaques is a hallmark of atherosclerosis; thus, we investigated the effect of the GDF-15 deficiency on the development and progression of the atherosclerotic lesions in the PT and BT. After 20 weeks CED, the lumen stenosis in the BT of GDF-15^−/−^ApoE^−/−^ mice was significantly (p < 0.001) reduced by 19.0% compared to ApoE^−/−^ mice and the lumen stenosis in the PT of GDF-15^−/−^ApoE^−/−^ mice was decreased by 6.7% compared to ApoE^−/−^ mice (Fig. 2A, B). Interestingly, comparison of the lumen stenosis between PT and BT within each genotype showed a significant (p < 0.001) 38.8% (ApoE^−/−^) or 26.4% (GDF-15^−/−^ApoE^−/−^) lower lumen stenosis in the PT compared with BT (Fig. 2A, B).

Additionally, we explored the necrotic core (Nc) formation in sections of BT and PT. We found in BT of GDF15^−/−^ApoE^−/−^ mice that decreased lumen stenosis was accompanied by a significant reduction of the Nc area (10%, p < 0.001) compared to ApoE^−/−^ (Fig. 2B, C); these morphological effects were also seen in PT with a significantly (p < 0.005) 6.5% reduced Nc area in GDF15^−/−^ApoE^−/−^ compared to ApoE^−/−^ mice (Fig. 2B, C). Moreover, when comparing BT vs PT, the percentage of Nc area in BP of ApoE^−/−^ mice was significantly (p < 0.04) 5.3% lower with no significant differences between Nc area in BT and PT of GDF-15^−/−^ApoE^−/−^ mice (Fig. 2C). Consequently, plaque vulnerability was analyzed (Additional file 2: Fig. S2): We found that in BT of GDF15^−/−^ApoE^−/−^ mice percentage of unstable lesions was 40% lower than in BT of ApoE^−/−^ mice; moreover, in PT of GDF15^−/−^ApoE^−/−^ mice percentage of unstable plaques was 57% lower than in ApoE^−/−^ mice (Additional file 2: Fig. S2). When comparing BT with PT, stable plaques are 23% more frequent in PT of GDF15^−/−^ApoE^−/−^ than in BT (Additional file 2: Fig. S2).

### (Immuno)histochemical characterization of atherosclerotic plaques

**Fig. 3:**
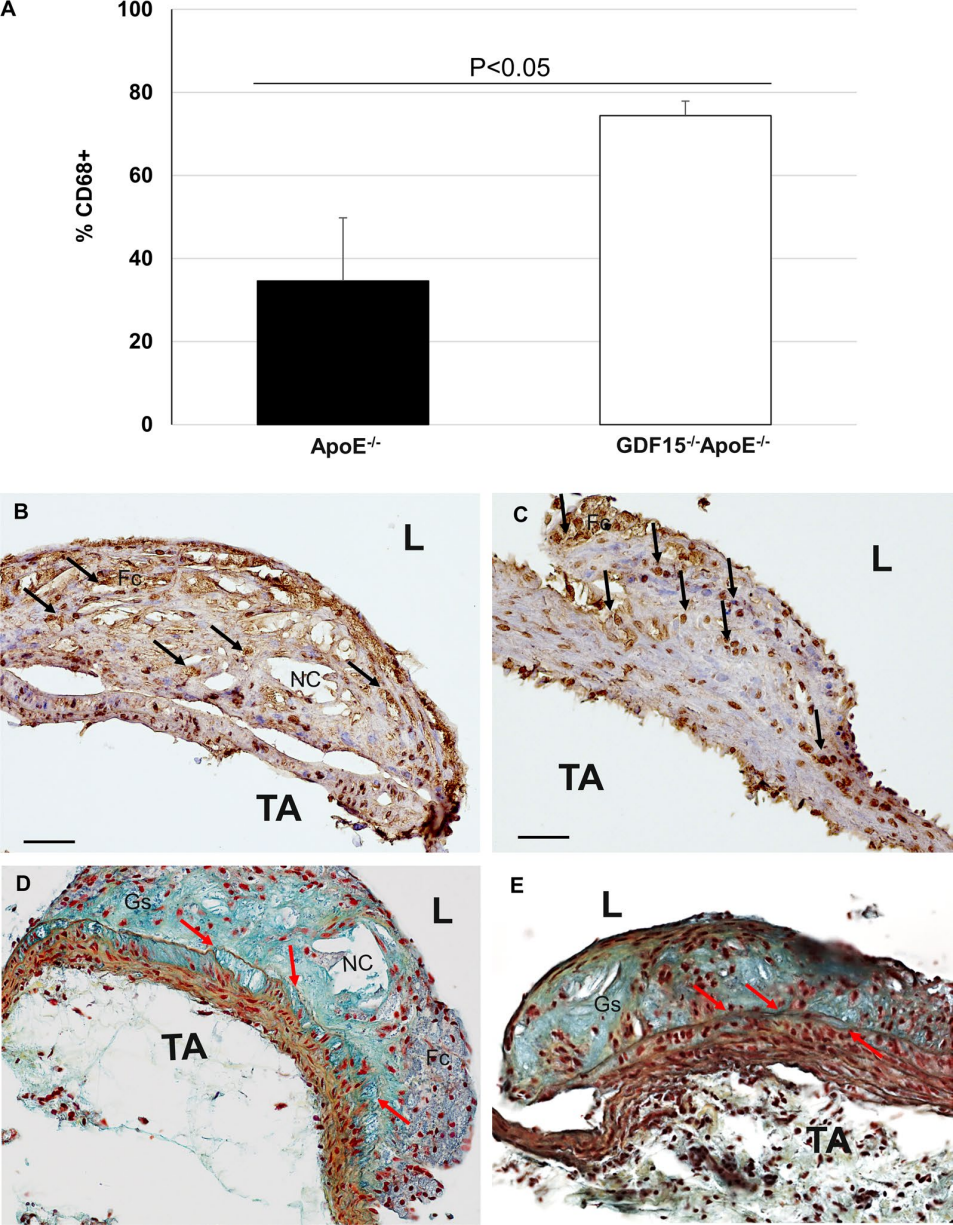
Effect of GDF-15 deficiency on morphology (MΦ, foam cells, collagen) of atherosclerotic plaques in the pulmonary trunk (PT). Immunohistomorphometric analyses of atherosclerotic lesions in the PT of ApoE^−/−^ and GDF15^−/−^ApoE^−/−^ mice after 20 weeks of CED. **A** CD68^ +^ -cells in atherosclerotic plaques, **B**, **C** representative CD68^+^ immuno-stained cross sections and (**D**, **E**) Movat´s stain, L: Lumen, Nc: Necrotic core; TA: Tunica adventitia. Co: Collagen (yellow); Fc: Foam cells; Gs: Ground substance (light blue); Black arrow: CD68^+^ MΦ. Red arrow: elastic lamina rupture. Data are expressed as mean + SEM; scale bar: 100 µm, n = 4–5

**Fig. 4:**
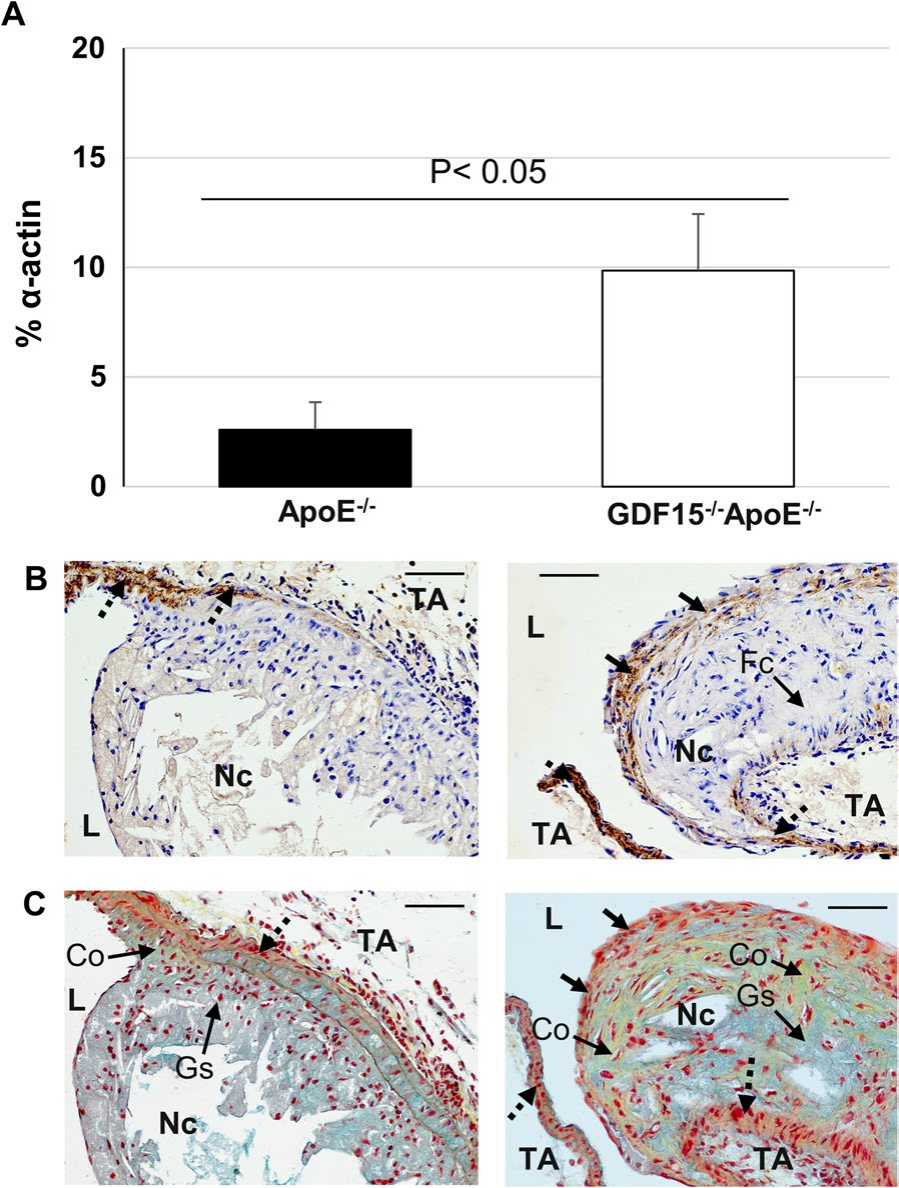
Effect of GDF 15 deficiency on the morphology (SMC, collagen) of atherosclerotic plaques in the pulmonary trunk (PT). Immunohistomorphometric analyses of atherosclerotic lesions in the PT of ApoE^−/−^ and GDF15^−/−^ApoE^−/−^ mice after 20 weeks of CED. **A** smooth muscle cells (α-actin^ +^) in atherosclerotic plaques. **B** representative α-actin^+^ immuno-stained cross sections and **C**  Movat´s stain. L: Lumen, Nc: Necrotic core; TA: Tunica adventitia. Co: Collagen (yellow); Fc: Foam cells; Gs: Ground substance (light blue); Nc: Necrotic core. Black arrow: SMC cap; black broken arrows: SMC within tunica media. Data are expressed as mean + SEM; scale bar: 100 µm; n = 4–5

**Fig. 5:**
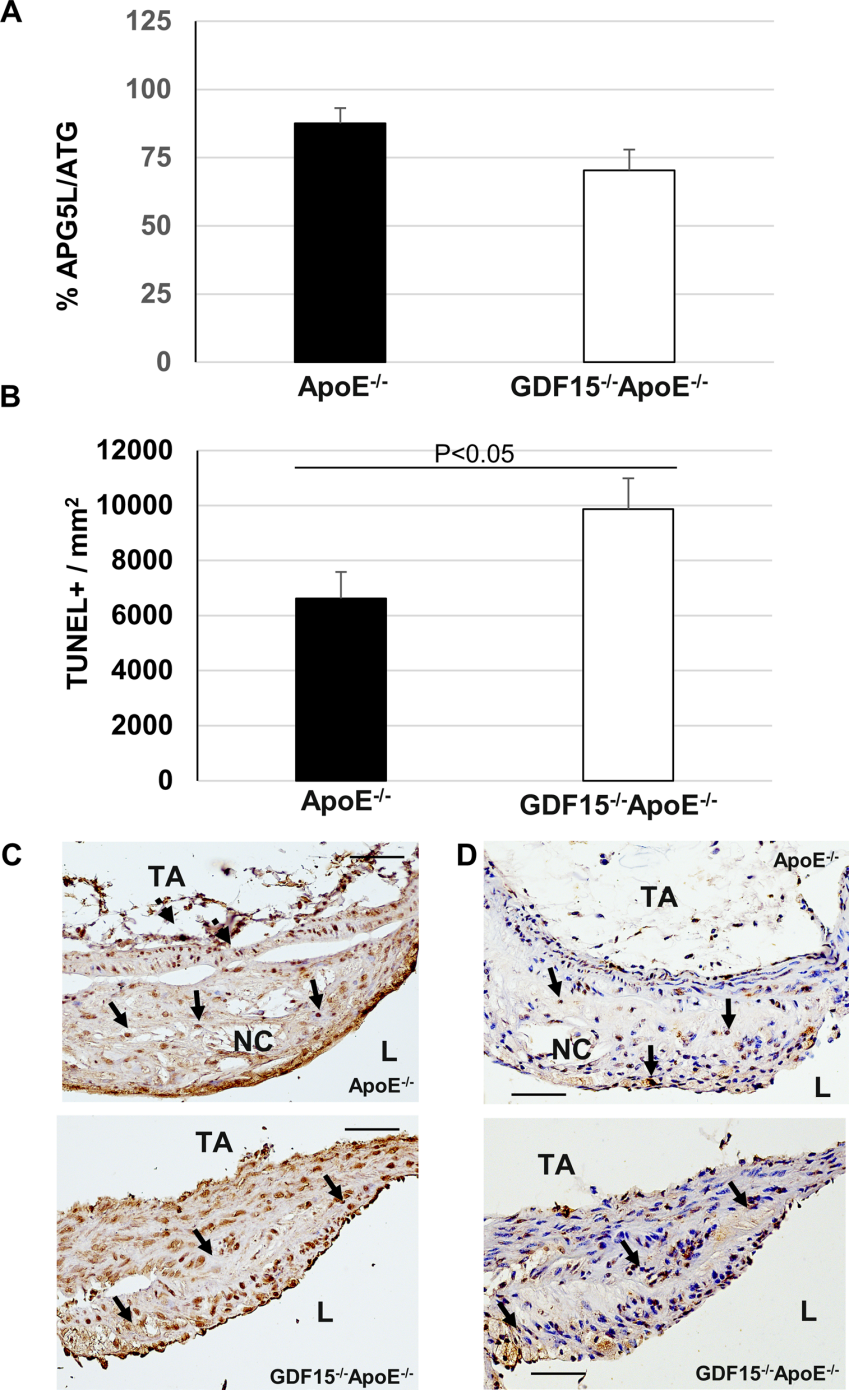
Effect of GDF-15 deficiency on the morphology (apoptosis, autophagy) of atherosclerotic plaques in the pulmonary trunk (PT). Immunohistomorphometric / histochemical analyses of atherosclerotic lesions in the PT of ApoE^−/−^ and GDF15^−/−^ApoE^−/−^ mice after 20 weeks of CED. **A** APG5L/ATG (autophagy); **B** TUNEL (apoptosis); representative pictures of immuno-/histochemistry reactions for **C** APG5L/ATG immuno-stained cross sections and **D** TUNEL^+^. Black arrow: APG5L/ATG^ +^ or TUNEL^+^; L: lumen; Nc: necrotic core; TA: tunica adventitia. Data are expressed as mean + SEM; scale bar 100 µm, n = 4–5

**Fig. 6:**
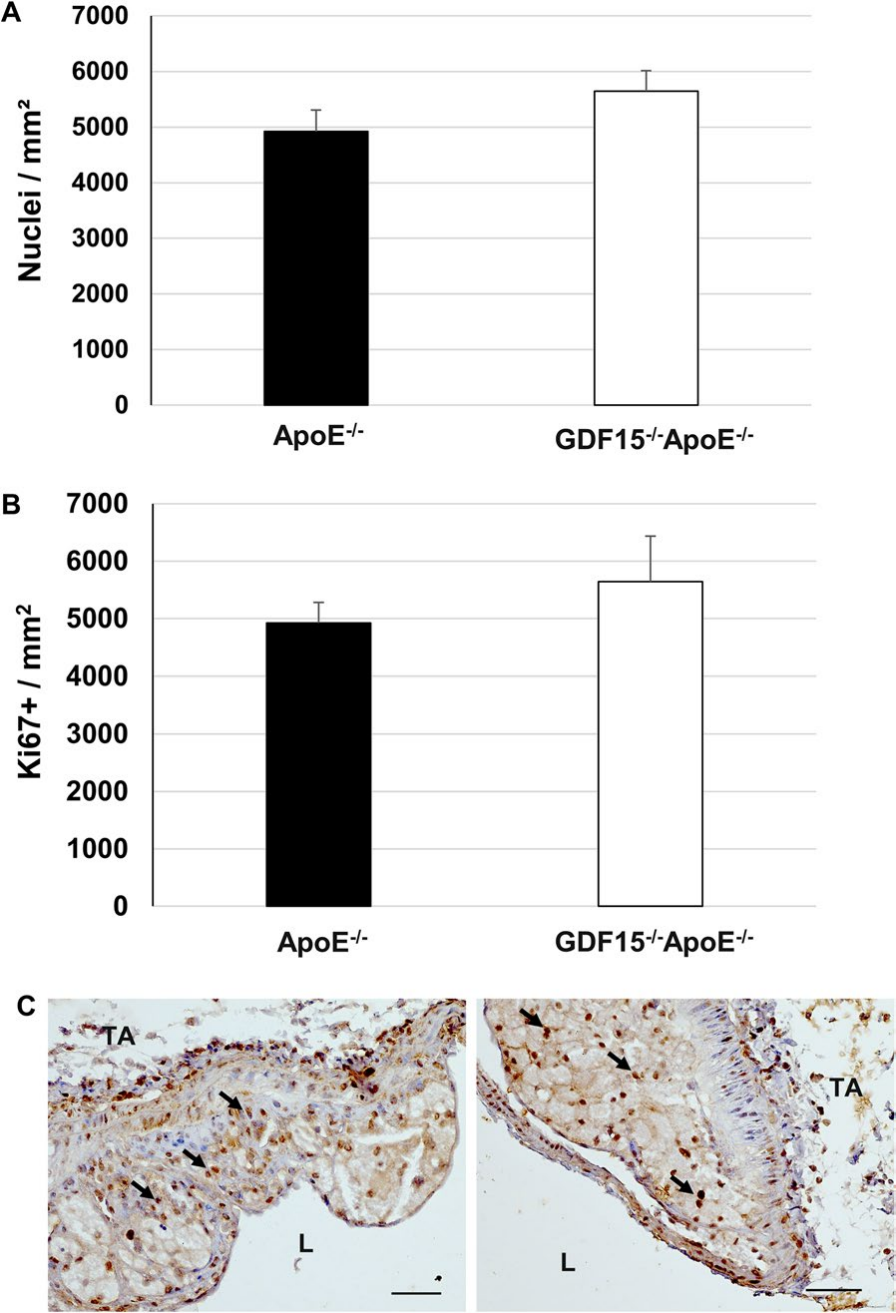
Effect of GDF-15 deficiency on the morphology (cell number, proliferation) of atherosclerotic plaques in the pulmonary trunk (PT). Immunohistomorphometric / histochemical analyses of atherosclerotic lesions in the PT of ApoE^−/−^ and GDF15^−/−^ApoE^−/−^ mice after 20 weeks of CED. **A** Cell density and **B** Ki67 (proliferation). **C** representative Ki67^+^ immuno-stained cross sections; data are expressed as mean + SEM; scale bar: 100 µm; n = 4–5

We determined the percentage of CD68^+^ MΦ in atherosclerotic lesions of the PT (Fig. 3). After 20 weeks of CED a significantly (p < 0.05) 39.8% higher percentage of CD68^+^ MΦ were found in plaques of GDF-15^−/−^ApoE^−/−^ mice compared with ApoE^−/−^ mice (Fig. 3A). The plaques of the PT in the mice of both genotypes showed compact structures and foamy morphology (Fig. 3B). Interestingly, blue/green colored proteoglycan is found as the predominant component in the neointima in lesions of ApoE^−/−^ and GDF-15^−/−^ApoE^−/−^ mice (Fig. 3C). A larger extension of the Nc areas can also be observed in the lesions of ApoE^−/−^ animals (Fig. 3C). The percentage of smooth muscle cells (SMC) is also an indicator of stable versus unstable plaques. In this context we found in atherosclerotic plaques in the PT of GDF-15^−/−^ApoE^−/−^ mice after 20 weeks CED that the percentage of α-actin^+^ SMC was significantly (P < 0.05) increased by 7.3% compared with ApoE^−/−^ mice (Fig. 4A). The sm-α-actin immunoreactivity and the MOVAT stain showed a cap-like localization in atherosclerotic plaques of both genotypes (Fig. 4B, C). Moreover, autophagy and apoptosis may also influence plaque stability. Thus, we investigated the density of APG5L/ATG^+^ cells in atherosclerotic lesions in the PT after 20 weeks of CED and found that the percentage of APG5L/ATG^+^ cells was 17.1% lower, whereas density of TUNEL^+^ cells was significantly (p < 0.05) 32.9% higher in GDF-15^−/−^ApoE^−/−^ than in ApoE^−/−^ mice (Fig. 5A-D). Additionally, after 20 weeks of CED in atherosclerotic plaques of the PT the cell density and percentage of proliferative Ki67^+^ cells were similar in both genotypes (Fig. 6A-C). We also investigated the localization of inflammatory cells and we found low immunoreactivities of COX-2 and IL-6 in the lesions of PT. However, when COX-2 and IL-6 positive reactions were observed, the location was found predominantly in the endothelial lining and the subendothelial space of PT in GDF-15^−/−^ApoE^−/−^ and ApoE^−/−^ mice, but not within the atherosclerotic plaque (Additional file 3: Fig. S3).

### (Immuno)histochemical characterization of atherosclerotic lesions in the human pulmonary artery

**Fig. 7:**
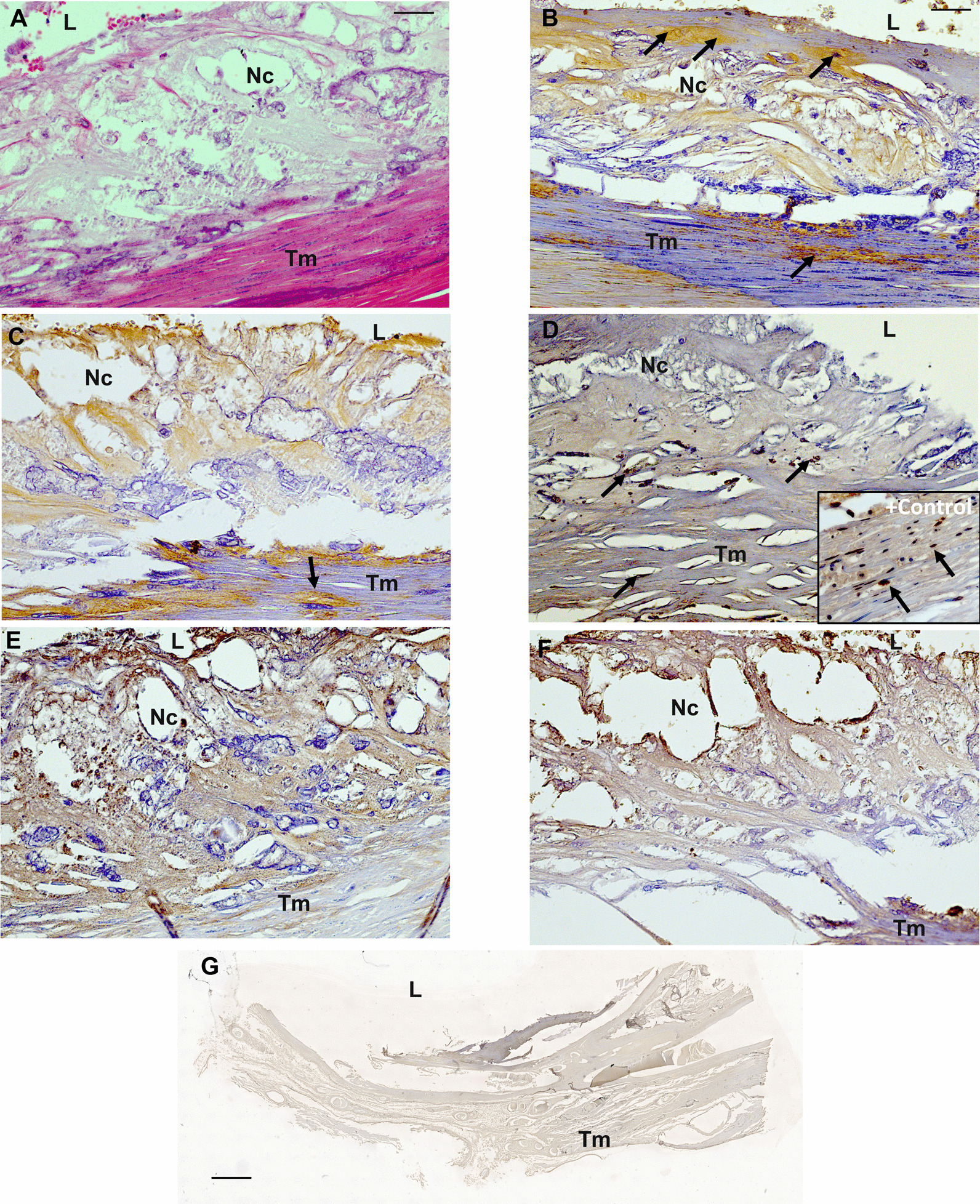
Exemplary post-mortem human necropsy of atherosclerotic pulmonary artery: **A** HE, **B** sm-α-actin^+^, (smooth muscle); **C** CD68^+^ (macrophages); **D** TUNEL (apoptosis); **E** Ki67 (proliferation), **F** APG5L/ATG (autophagy) and **G** negative control (absence of the primary antibody). Cell nuclei were counterstained with hematoxylin. L: Lumen, Nc: Necrotic core, Tm: Tunica media. Black arrow: positive immunoreactivity; magnification: scale bar 50 µm; negative control scale bar 100 µm

Because we asked whether these observations of atherosclerotic plaques in the PT only occur in mice, we further investigated human blood vessels with deoxygenated blood (pulmonary artery) including morphological examinations. In the human pulmonary artery (Additional file 1: Fig. S1), HE staining of atherosclerotic plaque showed the characteristic morphological alterations of the intima layer observed in other vessels with atherosclerotic lesions (Fig. 7A). The plaque mainly contained foam cells and a loose tissue matrix surrounding the cells and necrotic cores (Fig. 7A). Immunohistological investigations of the human atherosclerotic pulmonary artery using sm-α-actin antibodies showed, a cap-like coating of the plaque by SMC and fibrous cells (Fig. 7B). SMC also diffusely distributed within the plaque (Fig. 7B). Moreover, human atherosclerotic pulmonary artery showed CD68-immunoreactive MΦ (brown), demarcated from the remaining area (light blue) of the plaque and the vessel wall (Fig. 7C). Within the atherosclerotic plaque, MΦ revealed the characteristic form of foam cells. Additionally, TUNEL^+^ apoptotic cells, as well as proliferating Ki67^+^- or APG5L/ATG^+^ (autophagy) cells were observed to be distributed within the atherosclerotic lesion (Fig. 7D–F).

## Discussion

Atherosclerosis is characterized by a multifactorial pathophysiology that affects different organs, particularly the heart, brain, peripheral artery system and, thus, is associated with the development of CVD. In humans, atherosclerotic lesions usually are found at the origins of tributaries, bifurcations, and curvatures of arteries [49]. The nature of the disease could be explained by local disturbances in the blood flow leading to shear stress. The high and the low-shear areas have been considered as primary sites of atheroma formation in the arterial tree [50–52]. However, it has been frequently observed that atherosclerotic lesions do not develop in the veins in their normal environment of low pressure and a high flow. Interestingly, it has been shown that the veins develop atherosclerotic lesions when they are used as arterial bypass grafts where they are subjected to high pressure [5, 6]. Similarly, atherosclerotic lesions develop in the pulmonary arteries under pulmonary hypertension [33, 53]. Related to this, in general, high blood pressure is a well-recognized risk factor in CVD, a phenomenon that fits well in the hypothesis of “arterial wall stress” where the stress is produced not by blood flow but by blood pressure. In a similar way, low level of oxygen in the blood may contribute to the pathogenesis of various diseases of the vascular wall [28]. In this context, atherosclerosis in the pulmonary arteries, its branches, and after pulmonary hypertension are rarely affected by atherosclerosis and are not common in human [54, 55]. Most recently it has been shown that pulmonary artery calcification was significantly greater in patients with suspicion of stable angina pectoris compared to healthy control by using quantitative 18F-sodium fluoride positron emission tomography/computed tomography (NaF-PET/CT), a method that has been most recently suggested to be of clinical use in the early detection of pulmonary artery atherosclerosis [56]. In this regard, characterization of morphological differences between the lesions in pulmonary arteries and other vessels of the circulatory system may be of interest for early diagnosis and the use of different therapies. Recently, a sample of the pulmonary artery taken during the anatomy preparation course (Additional file 1: Fig. S1) has shown typical characteristics of advanced atherosclerotic lesions, with a cap-like coating of the plaque by SMC and fibrous cells, with SMC also diffusely distributed and extended foam cells within the plaque. Atherosclerotic processes analogously occurring in advanced human lesions are also seen in the BT of ApoE^−/−^ mice [57]. We found that long-term feeding of ApoE^−/−^ mice with CED is accompanied by up regulation of GDF-15 in atherosclerotic lesions, whereas GDF-15 deficiency reduced lumen stenosis in the BT as well as 18FDG uptake in the aortic arch [20]. However, not much is known about the influence of GDF-15 on the development of lesions in the pulmonary arteries or the PT. In this context, GDF-15 is involved in orchestrating atherosclerotic lesion progression by regulating apoptotic cell death and IL-6–dependent inflammatory responses to vascular injury [20]. GDF-15 deficiency inhibits significantly the lumen stenosis in the BT and the aortic arch compared with ApoE^−/−^ mice [20] as well as (by trend) in the PT, according to the present investigations. To characterize the morphological plaque composition in the PT, we performed (immuno)histochemical investigations. It is generally accepted that atherosclerotic lesions containing a high number of M2 MФ are more stable [58, 59]. In mouse, at the early stages of atherosclerotic plaques M2 MФ are present whereas M1 MФ are the most frequent phenotype in the advanced lesions [60, 61]. In this respect, our results show an increase of CD68^+^ in the GDF-15^−/−^ApoE^−/−^ compared to the ApoE^−/−^ mice in the PT supporting our previous work in which we have already demonstrated a similar effect in the BT [20].

However, we found that the percentage of the Nc area is lower in GDF-15^−/−^ApoE^−/−^ than in ApoE^−/−^ mice in BT and PT, a sign of instability in both kind of vessels. The presence of a lower density of MФ in the lesions of ApoE^−/−^ animals may be due to the fact that the Nc areas in this group are larger than in GDF-15^−/−^ApoE^−/−^ mice. These similar observations in both kind of vessels (PT and BT) indicate independence of the blood oxygenation and/or the pressure and appear to be affected by GDF-15 (deficiency) after CED. In this context, innumerable evidence confirms that endothelial dysfunction is a characteristic of patients with hypertension [62, 63]. Inflammation is a common mechanism related to endothelial dysfunction and there is a close relationship between oxidative stress and inflammation [64]. Consequently, we have investigated COX-2 and IL-6 immunoreactivities in the PT, as well as in the BT [20]. Unlike to our publication concerning BT [20] we here found a low COX-2 and IL-6 immunoreactivities in the lesions of PT were predominantly localized in endothelial cells and the subendothelial space of PT in GDF-15^−/−^ApoE^−/−^ and ApoE^−/−^ mice, but not within atherosclerotic plaque as we have found earlier in BT [20]. It is well known that the endothelial lining maintains normal with no or low expression of proinflammatory factors under normal homeostatic conditions. The well-known cardiovascular risk factors including smoking, aging, hypercholesterolemia, hypertension, etc. are associated with alterations in endothelial function. These characteristic findings and the lower degree of lumen stenosis observed in PT in comparison with BT could be related to the lower blood pressure in the PT. Thus, it is tempting to speculate that these circumstances inhibit the growth of lesions, leading to an improved stability in PT than in BT after 20 weeks of CED.

Recent evidence shows that SMCs contribute to the formation of the majority of atheroma foam cells in ApoE^−/−^ mice fed with a Western diet or standard chow for longer periods [65]. Sm-α-actin is an isoform typical of SMC, present in high amounts in vascular SMC and serves as a differentiation marker of SMC [66]. In previous publication, we showed no difference in the percentage of sm-α-actin–positive cells in atherosclerotic lesions in the BT in both genotypes [20], unlike what we found in the PT. In atherosclerotic plaques in the PT of GDF15^−/−^ApoE^−/−^ mice we observed an increase of CD68^+^ MΦ and sm-α-actin^+^ cells. However, since foam cells may originate from both, monocytes/MΦ or SMC, a characterization and identification of the origin of the foam cells can provide more information on the effect of GDF-15 (deficiency) concerning the effect of the oxygenation level or the blood pressure on the plaque development. Survival and death of MФ are important factors that affect the lesion development and progression. In this regard, we have previously shown that oxLDL induces GDF-15 expression and apoptosis in human MФ [44, 45]. Subsequently, we have shown for the first time that a consequence of GDF-15 deficiency results in inhibition of lumen stenosis in the BT of GDF15^−/−^ApoE^−/−^ mice after 20 weeks CED [20]. These effects were observed, regardless of inhibition of apoptosis as well as autophagy and an increase in cell density but without effect on proliferation [20]. However, compared with BT, in the PT we observed a similar effect on autophagy but not on apoptosis. In atherosclerotic plaques in the PT of GDF15^−/−^ApoE^−/−^ mice, it is likely that the increased apoptotic processes are responsible for the reduction of lumen stenosis, but without effecting cell density and, as in the BT, cell proliferation. This suggests that the PT has a different mechanism of lesion remodeling compared to the BT and the aortic arch, however, GDF-15 seem to be involved in this mechanism, too. In chronic inflammatory lesions, often under low oxygen concentrations, MΦ are abundant and adapted to this condition [67]. According to this, we found more CD68^+^ immunoreactivity in the PT of ApoE^−/−^ or GDF15^−/−^ApoE^−/−^ mice than in BT [20]. It has been described in human atherosclerotic lesions, that the hypoxic regions, e.g. the necrotic cores, are rich in foam cells and MΦ [68]. In this context, it is well known that in humans and animal models the growth of atherosclerotic plaques is accompanied by hypoxia, which promotes atherosclerosis [69, 70]. For many cell types, a major effect of hypoxia is the induction of apoptosis [71]. Hypoxia is a known stimulus of inflammation, angiogenesis, and apoptosis for MΦ [72]. MΦ begin to adopt a glycolytic metabolism allowing them to adapt readily when exposed to low oxygen conditions [67]. In this regard, it is proposed that certain populations of monocyte/MΦ survive better under conditions of low oxygen, thereby contributing to their increased numbers at sites of chronic inflammation as tumors, myocardial infarcts, and atherosclerotic plaques [67, 73]. This could be an explanation for an increased percentage of apoptotic cells and CD68^ +^ MФ in plaques of the PT than in the BT in both genotypes of mice. Despite the finding of an enhanced percentage of CD68^ +^ MФ in the PT compared to the BT, apoptosis levels are higher in the PT, suggesting that these apoptotic cells represent another type of cells, e.g. SMC or polarized MΦ. In vivo, in atherosclerotic lesions, the polarization of MΦ in M1 and M2 populations are increased during plaque progression, where also apoptotic cells are localized in rupture-prone areas and necrotic cores [72]. According to this, we found in lesions of the PT an increase of apoptosis in GDF-15 deficient mice accompanied by a reduction of the lumen stenosis. In this respect, hypoxia can induce survival or death by apoptosis or necrosis, depending on the cellular and metabolic environment [74, 75]. Related to this, GDF15 expression can increase in response to diverse extracellular stress signals, such as hypoxia/anoxia and inflammation [7], as described in cardiomyocytes, where GDF15 protects against apoptosis and protects the heart against ischemia/reperfusion [76]. The expression of GDF15 in cancer cell lines results in cell growth arrest and increased apoptosis, which suggests that GDF-15 may have antitumorigenic activity [7]. This may resemble the physiological hypoxic environment within atherosclerotic lesions, however, we found an increase of apoptosis in plaques of GDF-15^−/−^ApoE^−/−^ mice. Thus, it may be assumed that the balance or imbalance between proliferation, cell arrest, and apoptosis are critical factors in determining the plaque stadium and the development trend to a stable, unstable, or rupture-prone lesion. The specific response of the MΦ, polarization, apoptosis, necrosis, inflammation, perhaps depends on the ability of cells to adapt their metabolism to the plaque environment, e.g. hypoxia [73, 74]. However, studies related to atherosclerotic regression models propose that MΦ apoptosis is the major pathway for its removal from the plaque, although, recent studies have suggested that MΦ can proliferate in the arterial wall and the plaque. [77, 78].

Nevertheless, here we show for the first time a possible difference in the pattern of remodeling of the atherosclerotic lesion between the BT and PT in ApoE^−/−^, as well as in GDF-15^−/−^ApoE^−/−^ mice fed with CED and the importance of GDF-15 in this phenomenon. Inhibition of apoptosis may be anti-atherogenic and may thus be suggested as a therapeutic strategy to control plaque progression in the PT. Moreover, we have already shown that GDF-15 deficiency results in the activation of proapoptotic and at the same time, the induction of antiapoptotic genes in peritoneal MФ incubated with oxLDL in vitro [20]. These data are consistent with our in vivo findings in the BT, showing a reduction in TUNEL- or APG5L/ATG-positive cells in atherosclerotic plaques of GDF-15^−/−^ApoE^−/−^ mice [20]. These data confirm the assumption that GDF-15 may be important in MФ death and the remodeling of atherosclerotic lesions as has been also postulated by others [79–81] and that GDF-15 signaling may be a useful novel target for therapeutic intervention.

Atherosclerotic lesions in the pulmonary artery are frequently found in individuals with cardiopulmonary mechanisms of death, such as PE [55]. An early prognosis of PE is a crucial clinical challenge, which would allow choosing the appropriate treatment and reducing the mortality rate. In this context, GDF-15 has been identified as a predictor of CVD and related also to acute PE [82]. According to this, the serum level of GDF-15 was found to be significantly higher in patients with PE compared with controls [83, 84]. In this regard, we unexpectedly found in the human pulmonary artery from our anatomy course, a few numbers of GDF-15^+^ cells distributed, mainly in the tunica media (data not shown). This observation could be interpreted that the origin of circulating GDF-15 in patients with PE is probably not the pulmonary arteries.

GDF-15 expression can increase in response to diverse cellular stress signals, such as hypoxia/anoxia, inflammation, acute tissue injuries and tumoral processes [7]. In this respect, increased expression GDF-15—as observed in lungs of smokers and patients with COPD  contributes to cigarette smoke -induced pulmonary inflammation [85]. GDF-15 increases during COPD exacerbation but the role in stable COPD is unknown [26]. In this context, GDF-15 can be used as a systemic marker in patients with COPD, regardless of other cardiovascular risk factors [26]. Therefore, GDF-15 may be a powerful new biomarker not only for cardiovascular but also for cardiopulmonary vascular disorders as well as a therapeutic target.

## Conclusion

The size of the atherosclerotic lesions is smaller in PT than BT, possibly due to the effect of the low-oxygen blood and/or lower blood pressure. GDF-15 is involved in atherosclerotic processes in BT and PT, although different mechanisms in these two vessels seem to exist. In future studies measurements of the blood pressure as well as the blood oxygenation levels need to be performed to investigate a possible association between lumen stenosis and/or GDF-15 in low blood pressure vessels; characterization of MΦ subpopulations in atherosclerotic plaques should be done as well.”

## Limitations

Unfortunately, the blood pressure as well as the oxygenation levels were not evaluated in this study and thus, makes it not possible to investigate an association between lumen stenosis and/or GDF-15 in low blood pressure vessels. Moreover, regrettably the characterization of MΦ subpopulations was not performed, too.

## Supplementary Information

**Additional file 1.** **Figure S1**. Post-mortem human necropsy of atherosclerotic pulmonary artery: A) Left hilum of lung; B) Details of the pulmonary artery with atherosclerotic lesions (white star).

**Additional file 2.** **Figure S2**. Percentage of stable and unstable plaques in the PT of ApoE-/- and GDF15-/-ApoE-/- mice after 20 weeks of CED.

**Additional file 3.** **Figure S3**. Immunohistomorphometric analyses of atherosclerotic lesions in the PT of ApoE-/- and GDF15-/-ApoE-/- mice after 20 weeks of CED. Expression of the pro-inflammatory markers COX-2 (A and C) and IL-6 (B and D). L: lumen; Nc: necrotic core; TA: tunica adventitia. Black arrow: positive immunoreactivity; magnification: scale bar 100 µm.

## Data Availability

The datasets that support the findings of this study are available from the corresponding author on reasonable request.

## Abbreviations

ANOVA: Analysis of variance
APG5L/ATG: Autophagy Related 5
ApoE: Apolipoprotein E
BT: Brachiocephalic trunk
CED: Cholesterol-enriched diet
CHD: Coronary heart disease
COPD: Chronic obstructive pulmonary disease
CVD: Cardiovascular disease
CVS: Cardiovascular system
DAB: Diaminobenzidine
GDF-15: Growth differentiation factor-15
GPO-PAP: Glycerol- 3-phosphate oxidase–peroxidase
HIF-1: Hypoxia-inducible factor-1
HPR: Horseradish peroxidase
HUVEC: Umbilical vein endothelial cells
IPAH: Idiopathic pulmonary arterial hypertension
LDL: Low-density lipoprotein
LS: Lumen stenosis
LVH: Left ventricular hypertrophy
MΦ: Macrophage(s)
NaF-PET/CT: 18F-sodium fluoride positron emission tomography/computed tomography
oxLDL: Oxidized LDL
PAH: Pulmonary arterial hypertension
PBS: Phosphate buffered saline
PCR: Polymerase chain reaction
PFA: Paraformaldehyde;
PT: Pulmonary trunk
SEM: Standard error of the mean
SMC: Smooth muscle cells
TUNEL: TdT-mediated dUTP nick end labelling
