# Supplementary figures and images for: Characterization of atherosclerotic plaques in blood vessels with low oxygenated blood and blood pressure (Pulmonary trunk): role of growth differentiation factor-15 (GDF-15)
Source: BMC Cardiovasc Disord. 2021 Dec 17;21:601. doi: 10.1186/s12872-021-02420-9 (PMC8684150; doi:10.1186/s12872-021-02420-9)

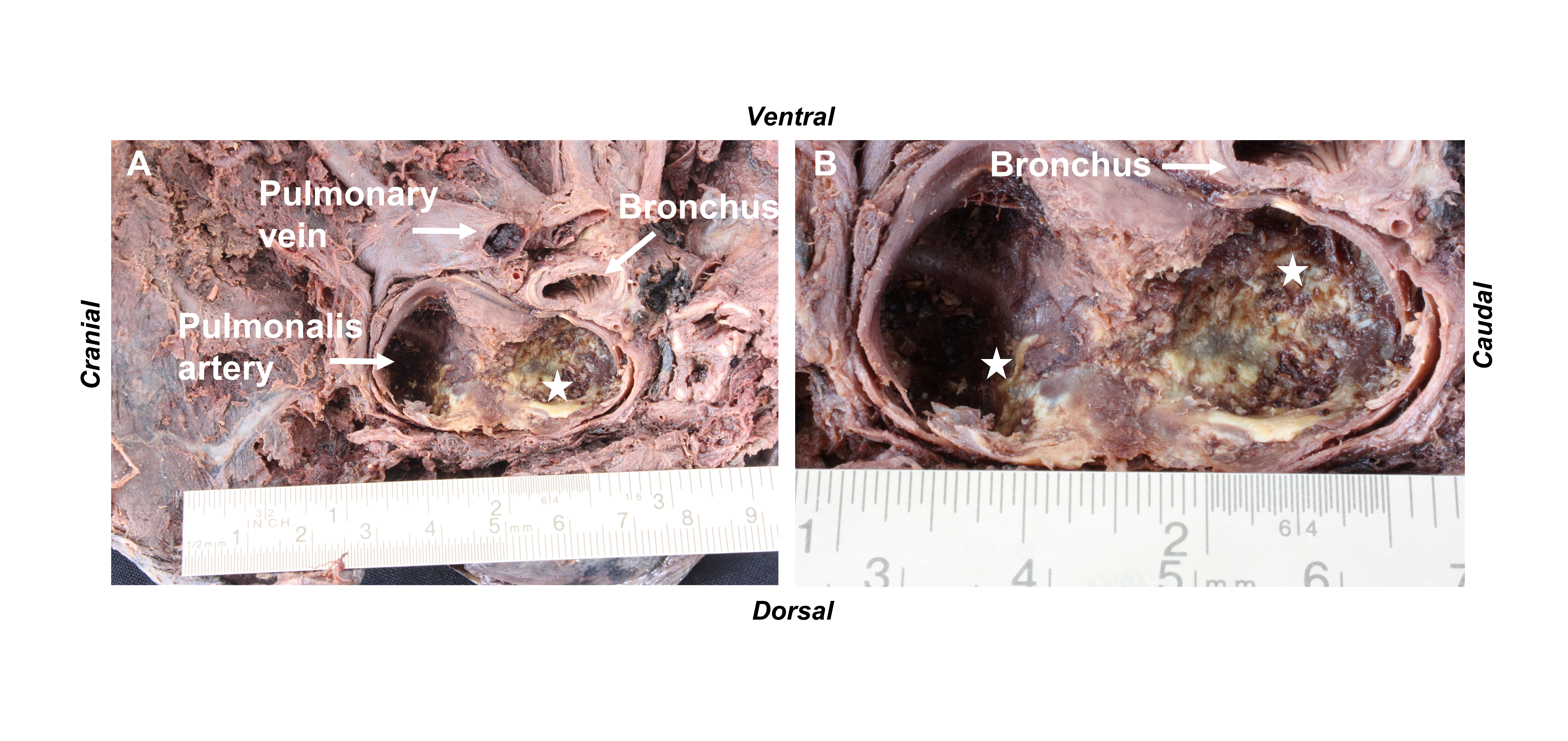

Supplement: Supplementary file 1 — Additional file 1. Figure S1. Post-mortem human necropsy of atherosclerotic pulmonary artery: A) Left hilum of lung; B) Details of the pulmonary artery with atherosclerotic lesions (white star). [file 12872_2021_2420_MOESM1_ESM.tif]

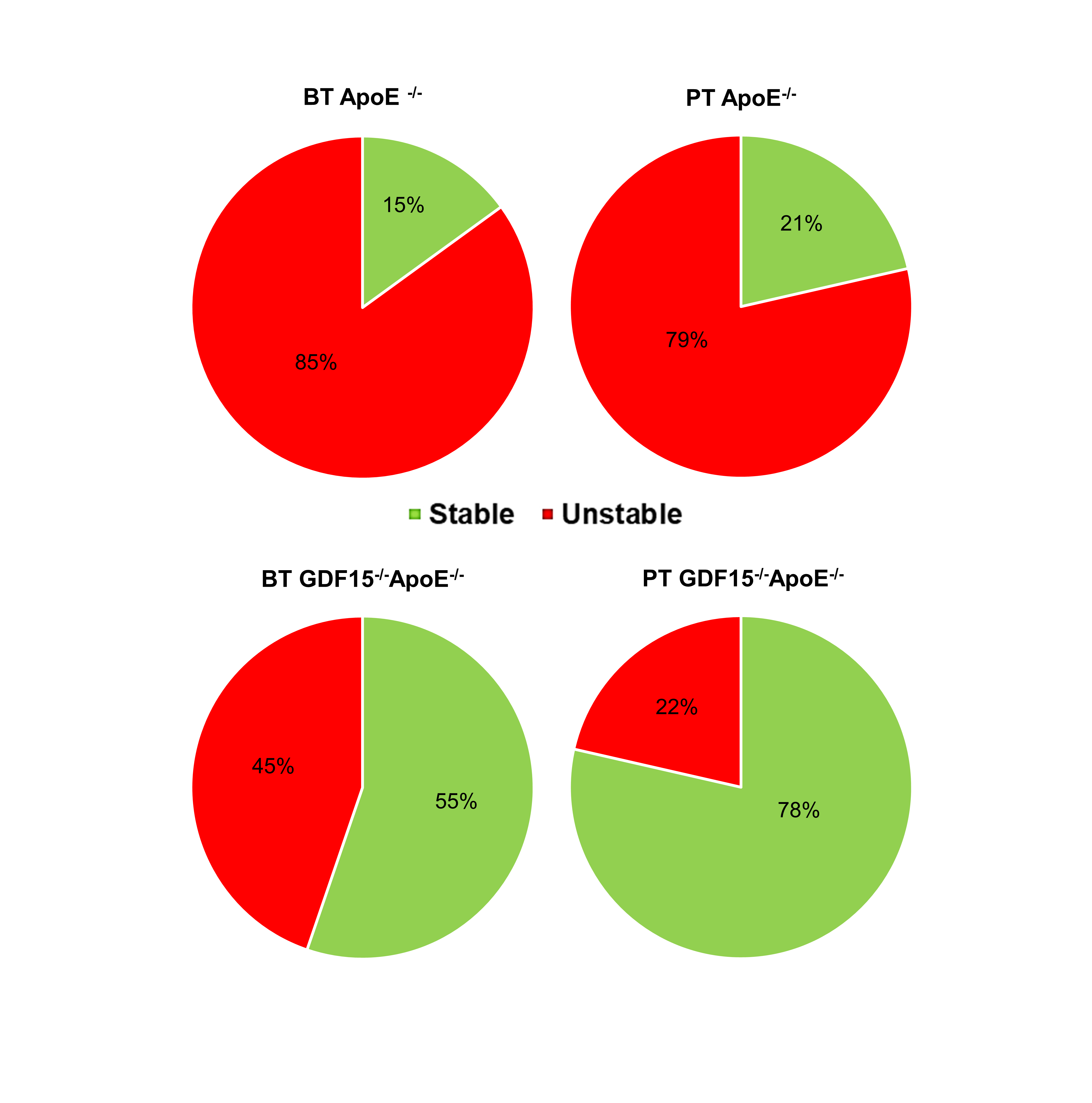

Supplement: Supplementary file 2 — Additional file 2. Figure S2. Percentage of stable and unstable plaques in the PT of ApoE-/- and GDF15-/-ApoE-/- mice after 20 weeks of CED. [file 12872_2021_2420_MOESM2_ESM.tif]

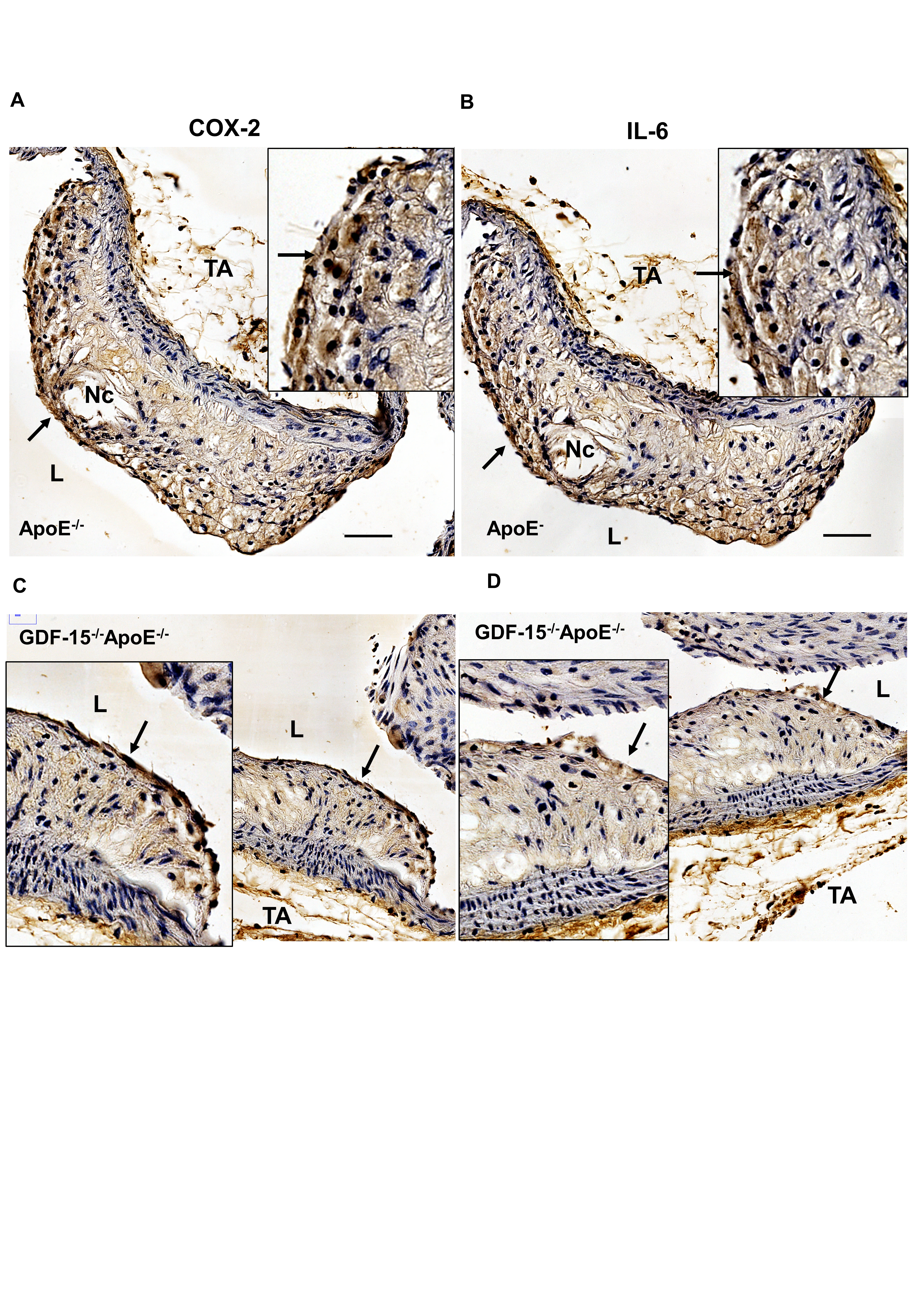

Supplement: Supplementary file 3 — Additional file 3. Figure S3. Immunohistomorphometric analyses of atherosclerotic lesions in the PT of ApoE-/- and GDF15-/-ApoE-/- mice after 20 weeks of CED. Expression of the pro-inflammatory markers COX-2 (A and C) and IL-6 (B and D). L: lumen; Nc: necrotic core; TA: tunica adventitia. Black arrow: positive immunoreactivity; magnification: scale bar 100 µm. [file 12872_2021_2420_MOESM3_ESM.tif]
